# Population Estimates of Self-Reported Depression and Anxiety in the US From a National Survey: Cross-Sectional Survey Study

**DOI:** 10.2196/70626

**Published:** 2025-04-16

**Authors:** Yuvraj Pathak, Elvira Makk-Frid

**Affiliations:** 1 West Health, 10350 N Torrey Pines Rd, San Diego, CA, 92037, United States, 1 858-535-7000

**Keywords:** depression, anxiety, survey

## Abstract

This letter shows that an estimated 31 million and 44 million adults self-report near-daily experiences of depression or anxiety, respectively. Of these, nearly a third have never spoken to a health care provider about it.

## Introduction

Mental health conditions, including depression and anxiety, have been on the rise in the United States over the past several years [1]. Data from the 2022 National Health Interview Survey indicate that the percentage of adults with anxiety and depression symptoms increased from 15.6% to 18.2% and 18.5% to 21.4%, respectively, between 2019 and 2022 [2]. Depression and anxiety can manifest as fatigue, difficulty concentrating, problems sleeping, or changes in appetite and negatively affect daily life [3,4].

Despite these impacts, people may not talk to their health care provider (HCP) due to stigma, cost, lack of access, or lack of insurance coverage [5]. In particular, stereotypes and prejudice can prevent people from discussing their mental health experiences with friends, family, or even HCPs, which could prevent them from receiving needed care [6].

In this paper, we estimate the number of people experiencing depression and anxiety almost daily based on a recent nationally representative survey. We also estimate how many of these people have never spoken to an HCP about their mental health. To the best of our knowledge, we are the first to report population-level estimates of self-reported experiences of depression and anxiety. While these results do not correspond to clinical diagnoses or a definite need for treatment, they provide timely information on an important issue and highlight the need to address mental and emotional health.

## Methods

### Ethical Considerations

The data for this cross-sectional study were obtained from a nationally representative West Health–Gallup web and mail survey conducted from November 2023 to January 2024; 5149 adults aged ≥18 years were sampled from the Gallup panel using stratified sampling spanning all 50 states and the District of Columbia. The survey response rate was 38%. Ethical approval was granted by Gallup’s internal institutional review board. Participation was voluntary, and responses were deidentified. No personal health information or other sensitive information was disclosed to the authors. Multimedia Appendix 1 provides details.

### Statistics

Percentages were estimated as the count of responses divided by the number of respondents, multiplied by 100. Probability sampling weights provided by Gallup were used to calculate the population estimates.

### Outcomes

Survey questions focused on perceptions about health care. Our 2 main outcomes were self-reported experiences of depression and anxiety. The survey asked respondents how many days they experienced either condition in the past 30 days and if they had ever discussed depression or anxiety with their HCP. Multimedia Appendix 1 provides more details.

## Results

**Figure 1.**
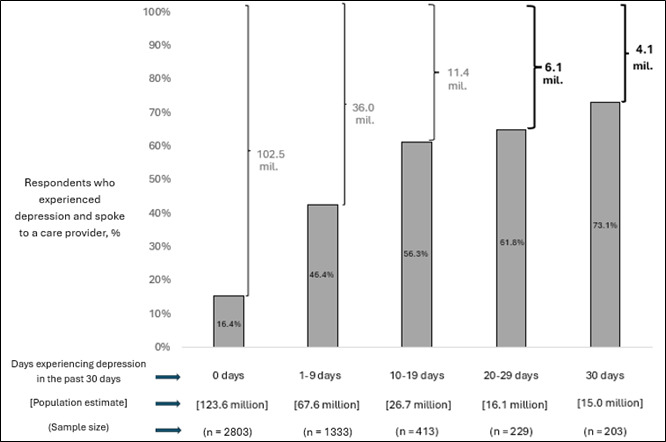
An estimated 10.2 million adults experienced feelings of depression almost every day but had never spoken to a health care provider about it. The figure plots the percentage of people who reported ever talking to a health care provider about depression on the y-axis against the number of days (category) they self-reported experiences of depression. The estimated population size and sample size, broken down across number of days reported (categorical variable), are displayed below the x-axis. The population estimates at the top of the figure, to the right of the curly brackets, represent the population estimates of the number of people who reported experiences of depression by number of days (categorical variable) but never talked to their health care provider about it. Percentages may not add up to 100 as some respondents may not have responded to one or both questions. Full details are available in Multimedia Appendix 1.

We estimate that 31.1 million US adults experienced symptoms of depression on 20 or more days in the past month at the time of this survey. Of these people, an estimated 10.2 million never spoke to an HCP about their mental health (Figure 1).

**Figure 2.**
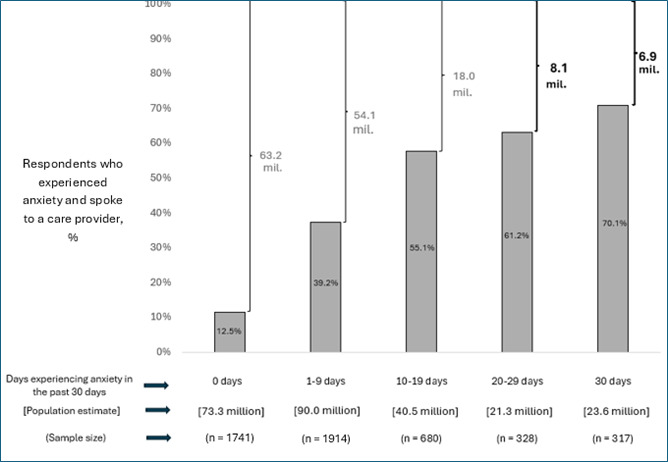
An estimated 15.0 million adults experienced feelings of anxiety almost every day but had never spoken to a health care provider about it. The figure plots the percentage of people who reported ever talking to a health care provider about anxiety on the y-axis against the number of days (category) they self-reported experiences of anxiety. The estimated population size and sample size, broken down across number of days reported (categorical variable), are displayed below the x-axis. The population estimates at the top of the figure, to the right of the curly brackets, represent the population estimates of the number of people who reported experiences of anxiety by number of days (categorical variable) but never talked to their health care provider about it. Percentages may not add up to 100 as some respondents may not have responded to one or both questions. Full details are available in Multimedia Appendix 1.

Additionally, we estimated that 44.9 million adults experienced anxiety on 20 or more days in the past month. Of them, an estimated 15 million had never spoken to an HCP about it (Figure 2).

## Discussion

### Principal Findings

We found that an estimated 53.8 million people have near-daily experiences of depression, anxiety, or both, and an estimated 18.6 million of them have never spoken to an HCP about these experiences.

### Limitations

Our findings are based on self-reported data, and we cannot assess if people (1) have a mental health diagnosis, (2) need professional care, or (3) have sought or received care. However, the findings provide useful population-level information about people’s self-reported experiences with depression or anxiety even though the results are not prevalence estimates.

### Comparison to Prior Work

The Substance Abuse and Mental Health Services Administration reports that 58.7 million adults had any mental illness in 2023 [7]. Based on the National Health Interview Survey, the US Centers for Disease Control and Prevention reports that 18.2% and 21.4% of adults experienced symptoms of anxiety and depression, respectively, in 2022. In comparison, we find that 53.8 million adults (18.5% of the adult population in 2024, as per author calculations) self-reported near-daily experiences of depression, anxiety, or both.

### Future Directions

This paper shows that millions of US adults experience depression, anxiety, or both on a near-daily basis but have never sought care from an HCP. The magnitude of these self-reported experiences, while alarming, does not correspond to a clinical diagnosis. Future research should aim to better understand the clinical needs of these people and build on the recent work showing a high degree of unmet need for mental health care [8].

## Supplementary material

10.2196/70626 Multimedia Appendix 1 Sampling strategy, list of questions, and detailed tabulations.

## Abbreviations

HCP: health care provider

## References

[1] Goodwin RD, Dierker LC, Wu M, Galea S, Hoven CW, Weinberger AH (2022). Trends in U.S. depression prevalence from 2015 to 2020: the widening treatment gap. Am J Prev Med.

[2] Terlizzi EP, Zablotsky B (2024). Symptoms of anxiety and depression among adults: United States, 2019 and 2022. Natl Health Stat Report.

[3] (2024). What is depression?. American Psychiatric Association.

[4] Worry and anxiety. US Centers for Disease Control and Prevention.

[5] Mongelli F, Georgakopoulos P, Pato MT (2020). Challenges and opportunities to meet the mental health needs of underserved and disenfranchised populations in the United States. Focus (Am Psychiatr Publ).

[6] (2024). Stigma, prejudice and discrimination against people with mental illness. American Psychiatric Association.

[7] (2023). Substance abuse and mental health services administration. US Department of Health and Human Services.

[8] Meiselbach MK, Ettman CK, Shen K, Castrucci BC, Galea S (2024). Unmet need for mental health care is common across insurance market segments in the United States. Health Aff Sch.

