# Supplementary material for: Population Estimates of Self-Reported Depression and Anxiety in the US From a National Survey: Cross-Sectional Survey Study
Source: Interact J Med Res. 2025 Apr 16;14:e70626. doi: 10.2196/70626 (PMC12017603; doi:10.2196/70626)

Sampling Strategy

This survey was fielded via web and mail to 5,149 adults aged 18 and older in all 50 states and the District of Columbia from November 13, 2023, to January 8, 2024, using the Gallup Panel. The mail portion of the survey was focused on older Americans aged 50 and older. The Gallup Panel is a probability-based panel of U.S. adults that is recruited using digital-dial (RDD) phone interviews and address-based sampling methods (ABS). At the conclusion of the RDD and ABS surveys, respondents are asked to agree to being re-contacted for future Gallup surveys. Approximately 80% agree and are eligible for recruitment onto the panel.

80,000 Panel members have provided necessary information to be contacted for web, mail, and telephone surveys. Another 20,000 members can be reached for mail and telephone surveys. receive an average of three surveys per month.

The average response rate on a Gallup Panel survey is approximately 40-45%. For this survey, the response rate was 38%. Web respondents were offered an incentive of $5 while mail respondents were offered an incentive of $2.

A stratified sample design was employed to collect the sample. 19 mutually exclusive strata were created, and samples were randomly selected from within each of the strata. Older adults age 50+ were oversampled to increase the stability of results for this population segment.

The sampling error is ±1.7% (±1.0 %) at 95% confidence interval for response percentages around 50 % (90%). Respondent knowledge and consent was obtained for all respondents and the final data was weighted to correct for non-response to match national level demographics as per Current Population Survey.

Questions

(note: the questions below were asked individually for each letter choice. Respondents provided separate answers for each option listed)

WH393 In the last thirty days, how many days did you experience the following?

1. Loneliness
2. Anxiety
3. Depression
4. Social isolation
5. Anger
6. Hopelessness

- 30 (1)
- 20 to 29 2)
- 10 to 19 (3)
- 1 to 9 (4)
- Zero (5)

WH393_2  Have you ever discussed the following with your primary care provider or other doctor?

1. Loneliness
2. Anxiety
3. Depression
4. Social isolation
5. Anger
6. Hopelessness

- Yes (1)
- No  (2)

**Detailed Tables**


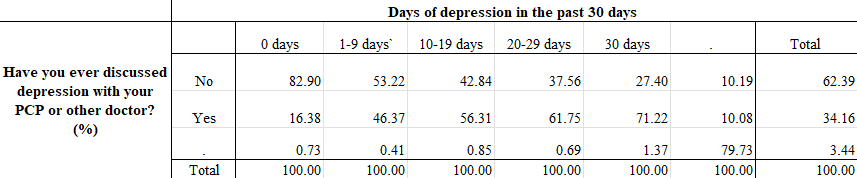
Percentage of people who have ever spoken to a provider about depression, by number of days they reported experiencing depression.

*
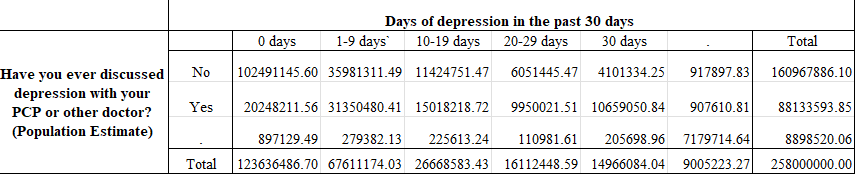
*Population estimates of people who have ever spoken to a provider about depression, by number of days they reported experiencing depression.


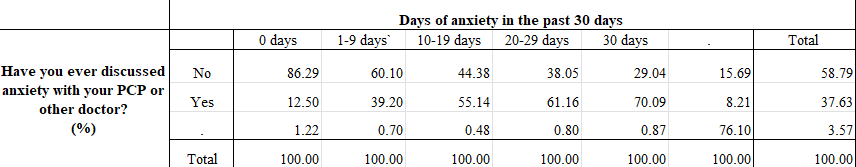
Percentages of people who have ever spoken to a provider about anxiety, by number of days they reported experiencing anxiety.

Population estimates of people who have ever spoken to a provider about anxiety, by number of days they reported experiencing anxiety.

*
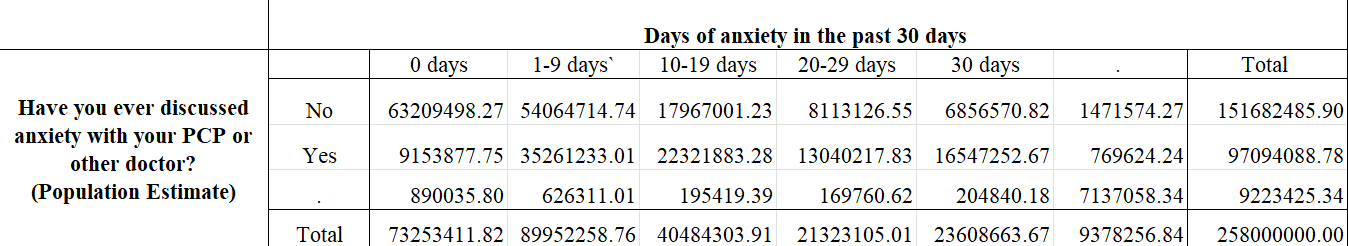
*

Percentage calculations of people who report near daily experiences of depression or anxiety but have never spoken to a provider about it.


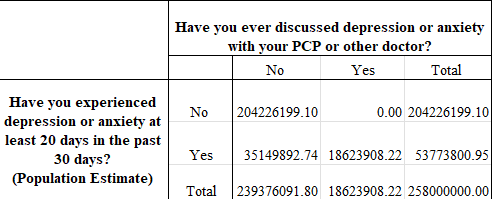

Supplement: Multimedia Appendix 1 [file ijmr-v14-e70626-s001.docx]
